# Surprising Structural and Functional Properties of Favism Erythrocytes Are Linked to Special Metabolic Regulation: A Cell Aging Study

**DOI:** 10.3390/ijms24010637

**Published:** 2022-12-30

**Authors:** Simone Dinarelli, Giovanni Longo, Stefka Germanova-Taneva, Svetla Todinova, Sashka Krumova, Marco Girasole

**Affiliations:** 1 Italian National Research Council (CNR), Institute for the Structure of the Matter (ISM), Via fosso del Cavaliere 100, 00133 Rome, Italy; 2 Bulgarian Academy of Sciences (BAS), Institute of Biophysics and Biomedical Engineering, G. Bonchev Str. 21, 1113 Sofia, Bulgaria

**Keywords:** erythrocytes, favism, cell aging, AFM, thermal stability of proteins, metabolic adaptation, membrane roughness, redox homeostasis

## Abstract

Favism uniquely arises from a genetic defect of the Glucose-6 Phosphate Dehydrogenase (G6PD) enzyme and results in a severe reduction of erythrocytes’ (RBCs) reducing power that impairs the cells’ ability to respond to oxidative stresses. After exposure to fava beans or a few other drugs, the patients experience acute hemolytic anemia due to RBCs’ lysis both intra and extra-vascularly. In the present paper, we compared selected biochemical, biophysical, and ultra-morphological properties of normal RBCs and cells from favism patients measured along cellular aging. Along the aging path, the cells’ characteristics change, and their structural and functional properties degrade for both samples, but with different patterns and effectors that have been characterized in biophysical and biochemical terms. In particular, the analysis revealed distinct metabolic regulation in G6DP-deficient cells that determines important peculiarities in the cell properties during aging. Remarkably, the initial higher fragility and occurrence of structural/morphological alterations of favism cells develop, with longer aging times, into a stronger resistance to external stresses and higher general resilience. This surprisingly higher endurance against cell aging has been related to a special mechanism of metabolic regulation that permits lower energy consumption in environmental stress conditions. Our results provided a direct and coherent link between the RBCs’ metabolic regulation and the cell properties that would not have been possible to establish without an investigation performed during aging. The consequences of this new knowledge, in particular, can be discussed in a more general context, such as understanding the role of the present findings in determining the characteristics of the favism pathology as a whole.

## 1. Introduction

Favism is the most widespread pathology known, which arises from an enzymatic defect and has an estimated spread of approximately 400 million people worldwide [1].

Even though this pathology has been known for many centuries, it has long been interpreted as an allergy, and it was only in the 20th century that its real genetic base was finally discovered [2]. The enzymatic defect that causes the disease involves the Glucose-6-Phosphate Dehydrogenase (G6PD), a key housekeeping enzyme whose impairment makes the cells highly deficient in reducing power (namely, nicotinamide adenine dinucleotide phosphate, NADPH) and very susceptible to oxidative stress. This is particularly serious and evident in the case of red blood cells (RBCs). Favism is indeed characterized by hemolytic crises, which can be very severe and occur when the patient is exposed, usually by ingestion, to fava beans or a few other drugs or chemicals [3]. In the absence of the exogenous agent, the patient is asymptomatic and, sometimes, can even ignore his pathological condition.

Over the years, the biochemical pathway leading to hemolytic crises has been characterized in good detail: Following the ingestion of fava beans, the contained vicine and convicine are converted into divicine and isouramil. These latter molecules have high redox activity and are very effective in producing reactive oxygen species (ROS), [4,5], especially hydrogen peroxide and the super-oxide anion. In normal RBCs, hydrogen peroxide is cleared by means of the intracellular catalase and glutathione peroxidase [6], which require NADPH. However, in favism patients, the erythrocytes have strongly reduced production of NADPH, and they cannot effectively reverse glutathione consumption. As a consequence of the weakness of these antioxidant systems, RBCs undergo severe oxidative damage, in particular at the level of the membrane lipids and proteins, including the most important intracellular protein, hemoglobin (Hb), which promotes the degradative paths. The most severely damaged erythrocytes hemolyze both intravascularly and extra-vascularly with a pattern that is also associated with hyperbilirubinemia [7]. A significant part of the damaged RBCs, however, is removed from the blood circulation by macrophages as “aged” cells [8]. The most damaged cells are typically the more senescent, as it is assumed that the G6PD and the antioxidant systems’ activity further degrade throughout the cell lifespan.

Genetically speaking, the gene encoding for G6PD is located on the long arm of the X chromosome, and the pathology is transmitted as an X-linked inherited trait. The most severe mutations affect the enzyme stability in the cells such as those involving residues at the dimer interface or those that interact with a structural NADP molecule [9].

The symptoms of favism depend on the genetic mutation of G6PD, but also on the dose of the exogenous stimulus [10,11]. Regarding the first, over 400 mutations of G6PD are known, associated with different residual activity of the enzyme. The mutant G6PDs are distributed territorially in a fairly homogeneous way, likely as a consequence of the protection provided against malaria. This latter circumstance has indeed likely played a role in the diffusion and territorial stabilization of the mutant G6PD. The characteristics and severity of the symptoms are different in the tropical African (the most diffuse) and North American variants (especially spread in the Afro-American community) compared to the Mediterranean, tropical Asian, or the other different forms of this disease.

Concerning the dependence of symptoms on the dose of the exogenous agent, it was observed that, in vivo, the larger the initial dose, the greater the hemolytic crisis. This occurs because the symptoms are connected to cellular aging. Indeed, in the presence of a hemolytic stimulus, the first cells to lysate are the oldest, in which the reducing power, or enzymatic activity, has progressively decreased [12]. To underscore this effect, the administration of a second, identical dose of the exogenous agent causes a second crisis much milder than the first one.

The dependence of the symptoms on the RBCs’ age is particularly interesting for us because, in recent years, we have developed an in vitro experimental model based on the artificially accelerated aging of RBCs. The model system allows one to analyze, in detail, the biochemical, metabolic, and structural characteristics of erythrocytes according to the stage of aging and the possible administration of external stimuli. For example, the characteristics of erythrocyte aging in native conditions, in microgravity, and in the presence of mechanical stimuli [13,14,15] were analyzed.

From a methodological point of view, our present protocol involves the evaluation of biochemical, biophysical, and morphological-structural properties of the cells by combining conventional biochemical assays to determine the main cellular metabolic parameters, differential scanning calorimetry (DSC) for the evaluation of the structural stability of the main cellular components, and accurate morphological assessment using atomic force microscopy (AFM). Indeed, the excellent resolution provided by AFM imaging also allows us to unequivocally discern the biomolecular morphologies by accurate volumetric assays [16], and previous experiments involving AFM characterization of RBCs have shown that this technique is particularly suitable to reveal the alterations of the RBCs’ plasma membrane that take place during aging. In particular, measurement of the membrane roughness was used to highlight the state of the structural integrity of the membrane-cytoskeleton, with particular regard to the mechanical support exerted by the skeleton on the membrane. In fact, previous data have shown that the roughness of the plasma membrane of RBCs is significantly sensitive to structural alterations (e.g., membrane–skeleton detachments) occurring during aging or as a result of genetic defects [17,18].

In this work, we present a comparison of the characteristics of human RBCs from a healthy donor and favism patients during aging. The rationale of the study is to investigate the occurrence of critical phenomena that, during the senescence pathway, allow one to distinguish the behavior of pathological cells from normal ones and highlight a correlation between the behavior of these cells and their structural and biochemical characteristics. In this framework, the data presented here reveal remarkable biophysical and morphological properties of the G6PD-deficient cells that, coupled with a peculiar metabolic regulation, make these cells significantly more fragile at lower aging times but much more resilient to aging-induced alterations further down the line compared to the normal erythrocytes.

## 2. Results

**Figure 1:**
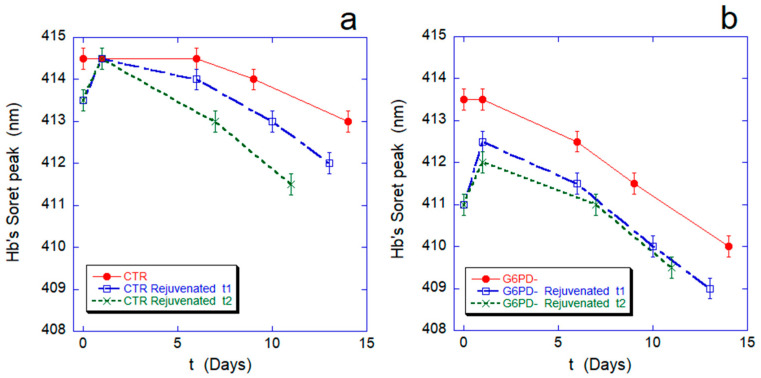
Hb oxidation rate along the RBCs aging for normal cells (panel (**a**)) and G6PD-defective erythrocytes (panel (**b**)). In each panel, the red curve is the oxidation curve on the native sample, while the blue and green lines refer to rejuvenation performed after 6 (rejuvenated t1) or 12 days (rejuvenated t2). The Hb oxidation curves after revitalization are artificially shifted to zero (relative time) for ease of comparison. All data points are reported with their standard deviation (SD).

**Figure 2:**
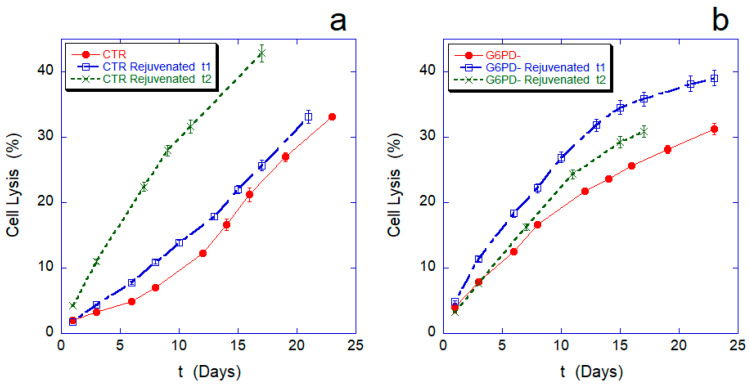
Cell lysis percentage measured during aging for normal (panel (**a**)) and G6PD-defective erythrocytes (panel (**b**)). In each panel, the red curve is the cell lysis on the native sample, while the blue and green lines refer, respectively, to rejuvenation performed after 6 (rejuvenated t1) or after 12 days (rejuvenated t2). In the graphs, the lysis curves after revitalization have been artificially shifted to t = 0 (relative time) for ease of comparison. All data points are reported with their SD.

Figure 1 and Figure 2 show the hemoglobin oxidation rate and the lysis curves measured, respectively, during aging for both the control sample and the cells defective in G6PD. For a more in-depth investigation, we compared these data with the curves obtained after revitalization processes performed after 6 (rejuvenated t1) or 12 days of aging (rejuvenated t2), respectively. The purpose of the rejuvenation process is to allow metabolically active cells to reactivate metabolic synthesis for the production of ATP and, possibly, reducing power, through the administration of the elementary building blocks. The process, on the one hand, determines an energetic re-synchronization of the cells, but on the other, in fact, eliminates from the sample the cells that are unable to actively run their energy metabolism.

The comparison between the dynamics of Hb oxidation in normal and pathological cells (Figure 1a,b, red curves) confirms a faster and more severe oxidation rate in G6PD-deficient cells.

After rejuvenation performed after 6 or 12 days, the curves show an additional 1-day reduction of the Fe-Hb, which was already described [14] and is due to the long-term effect of the reload in ATP and reducing power. This transient phase is followed by progressive re-oxidation, with the Soret peak moving towards a shorter wavelength.

After the revitalization performed after 6 or after 12 days, the defects in the redox machinery of the favism erythrocytes result in an incomplete reduction of Hb (412.5 nm instead of 413.5 nm, which is, in any case, lower than the 414.5 nm of the controls) while different re-oxidation rates can be observed: Accelerating with aging time in the controls while essentially identical to the native red curve in the pathological cells. These sharp differences in behavior suggest that different metabolic regulations take place in the two systems.

Concerning cell lysis (Figure 2), a natural criterion for cell death could be to assume that cell lysis occurs when the intracellular volume increases up to a level that cannot be sustained by the cell [19]. In our experiments, the dynamics of cell lysis observed in the two samples were very different, both for the trend of the initial parts of the curves and for the behavior of the lysis after the revitalization. Indeed, the controls have good resistance to lysis in the first 7–8 days, after which the lysis rate (tangent of the lysis curve) increases significantly; in contrast, G6PD-cells are much more fragile than the controls in the first 10–12 days, but at longer durations, their lysis rate decreases significantly. The point of intersection of the lysis curves for the two samples occurs at approximately 30% of total lysis, i.e., after approximately 22/23 days. Therefore, cells deficient in G6PD are initially more susceptible to lysis, but over time, they show resistance to aging greater than the controls.

These differences even appear amplified in the lysis curves measured after revitalization.

In general, and in agreement with previous measurements, the lysis curves of the revitalized controls have a higher slope, and the lysis rate increases over time and with the day on which the revitalization was performed (e.g., rejuvenated t2 > rejuvenated t1). Remarkably, however, this is not true for the sample defective in G6PD. In this case, the curve after the first revitalization shows much more fragile cells than the basic lysis curve, but in the second order of revitalization (performed after 12 days of aging), the residual cells are much less fragile than before. Although these trends are, qualitatively speaking, in agreement with what was observed in the basic lysis curve, this constitutes a significant result because a similar behavior has never been observed before and it indicates the existence of a significant fraction of cells with a strong resistance to the lysis. Clearly, this resistant fraction of RBCs characterizes the sample behavior at very long aging times.

The explanation for these unusual resistances to lysis could depend, in principle, on intrinsic structural differences, or it could be caused by different metabolic characteristics or adaptations, that take place in G6PD-defective cells and that distinguish their behavior from the controls. To elucidate this point, we explored some aspects of the structural, morphological, and metabolic characteristics of the samples.

**Figure 3:**
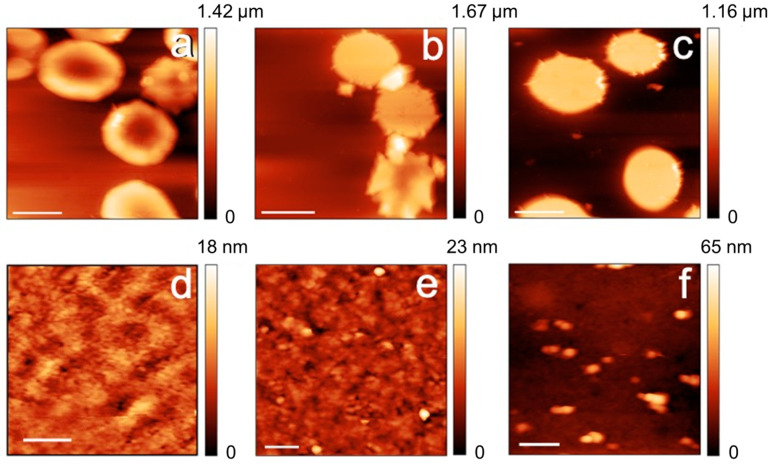
AFM images of the cell morphologies (panels (**a**–**c**); scale bars 5 μm) and high-resolution images of the plasma membrane (panels (**d**–**f**); scale bars 400 nm). The images refer to control RBCs imaged after 1 day (**a**,**d**), 7 days (**b**,**e**), and 12 days of aging (**c**,**f**).

**Figure 4:**
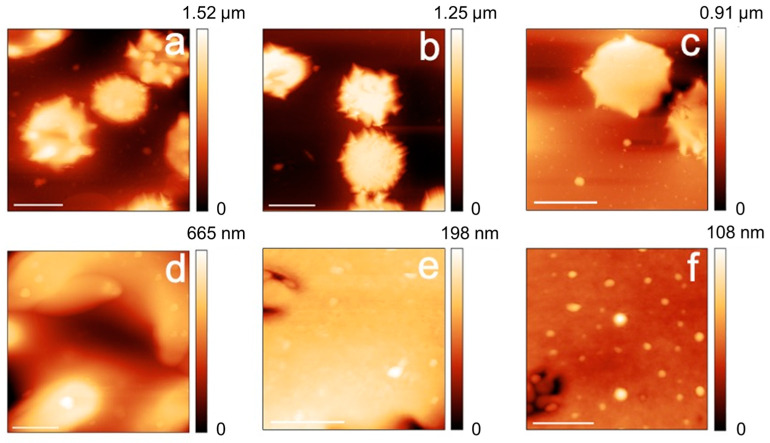
AFM images of the cell morphologies (panels (**a**–**c**); scale bars 5 μm) and high-resolution images of the plasma membrane (panels (**d**–**f**); scale bars 400 nm). The images refer to G6PD deficient erythrocytes imaged after 1 day (**a**,**d**), 7 days (**b**,**e**), and 12 days of aging (**c**,**f**).

The morphological (AFM) data, reported in Figure 3 and Figure 4, show that both the control and the cells defective in G6FD develop micro-vesicles and, at longer aging times, extensive vesiculation, which are both well-known markers of cell aging [20,21]. Interestingly, the G6PD-defective cells produce these vesicles since the beginning [8] and, certainly, with a higher abundance compared to the control samples. In addition to vesiculation, other morphological markers of aging, such as the presence of spherocytes or the presence of proto-spicules on the membrane, are also observed at shorter aging times or are more abundant in G6PD-defective samples. Overall, therefore, compared to controls, the aging pathway of defective cells in G6PD is, in morphological terms, more severe and more accelerated in its initial phase (i.e., short aging times). On the other hand, it should be noted that the morphological characteristics of the pathological cells remain, mostly, unaltered during aging, and in contrast, a relevant and progressive evolution of the aging markers (e.g., percentage of swollen or spiculed cells, development of micro-vesicles, etc.) takes place in the control samples.

In addition to the purely morphological data, it is interesting to analyze the trend of the changes in the plasma membrane roughness. This latter parameter is sensitive to the membrane arrangement, as well as to the structural integrity and the mechanical support exerted by the RBC skeleton on the overlying membrane [17].

**Figure 5:**
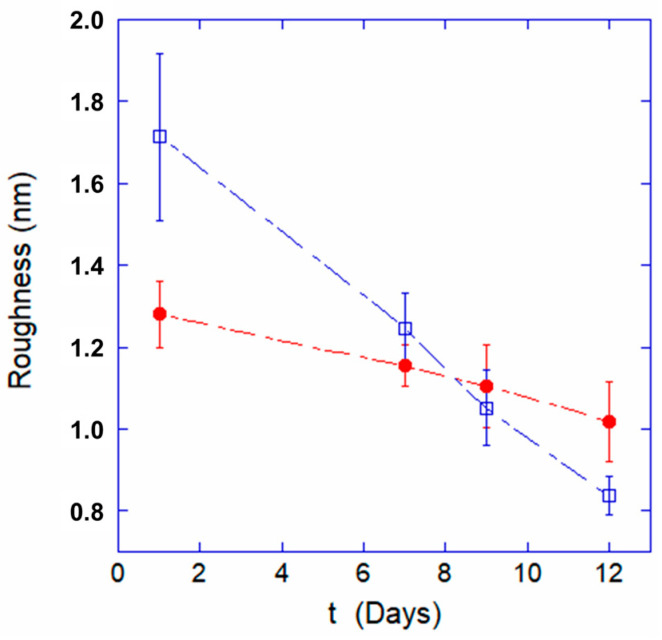
Variation of the plasma membrane roughness at increasing aging time for normal cells (open blue squares) and G6PD-deficient cells (red circles).

The roughness data, reported in Figure 5, show substantial differences between the two samples. Indeed, despite both trends monotonously decreasing, the quantitative value of the membrane roughness for the G6PD-defective cells is much smaller already after 1 day, and the decreasing rate is much slower than for the control cells. As a consequence, the roughness at the 12-day endpoint is higher in the G6PD-deficient samples than in the controls. The roughness trends highlight an extremely intriguing feature of G6PD-defective erythrocytes that somehow confirm what was observed in the morphological data: They appear to have a worse initial health status but they are much more resilient and endure aging better than the control cells.

Since morphological trends, with special regards for the membrane roughness, have been associated in the past with the concentration of intracellular ATP, it is interesting to quantify, over time, the concentration of this fundamental energy resource [14,21].

**Figure 6:**
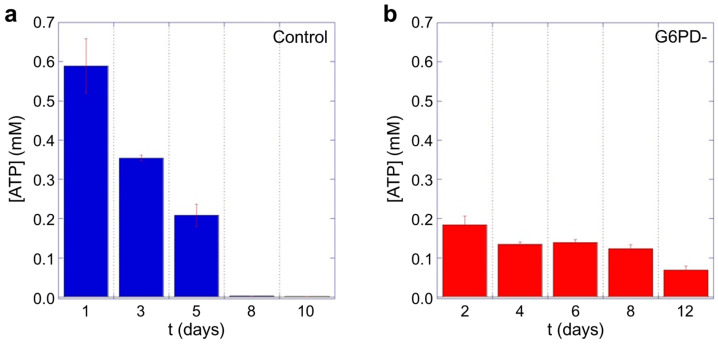
Intracellular ATP measured as function of aging time for (**a**) control cells and (**b**) erythrocytes from favism patient. Data are in linear scale and evidence a remarkable difference both in the initial concentration and in the ATP consumption rate over time. An ANOVA test confirms that the differences between the corresponding CTR and pathological data are statistically significant (*p* < 0.05).

The data shown in Figure 6 highlights significant differences between the controls and the samples defective in G6PD. For example, at short aging times, the intracellular concentration of ATP in the favic erythrocytes is much lower (at least 3 times) than the controls. Yet, the consumption of ATP over time in the pathological cells results in being extremely small. Consequently, at long aging times (>7/8 days), while the controls are in a condition of critical energy deficiency, the cells deficient in G6PD still retain a significant amount of ATP available for use, thus determining important behavioral, and possibly structural, differences at these timepoints. Moreover, it should be noted that the cells defective in G6PD have initial concentrations of ATP much smaller than those of the control erythrocytes and this is expected to determine consequences both on the structural stability and on functional characteristics of the native pathological RBCs.

Finally, it is worth noting that the characteristics of the ATP trend measured for the control samples are fully compatible with previously reported data [14,15] measured on different healthy donors.

To gain more information on the structural stability of these samples, we performed an analysis of the samples by DSC and evaluated their osmotic resistance.

**Figure 7:**
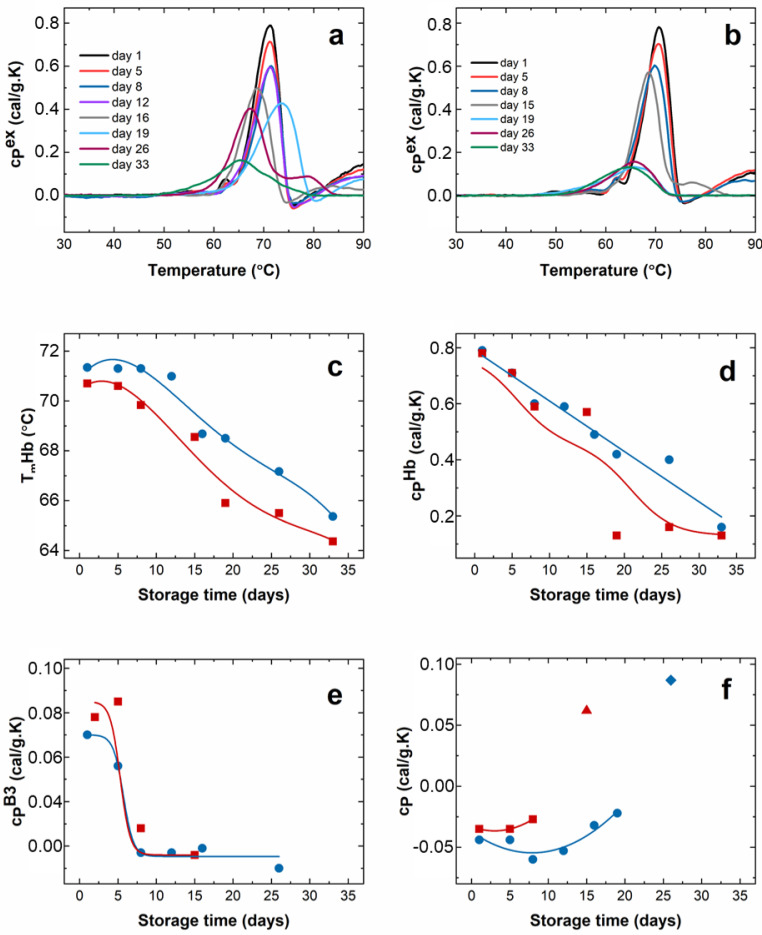
Calorimetric characteristics of RBCs: Calorimetric scans recorded during the cells aging for a period of 33 days for healthy (**a**) and G6PD-defective (**b**) subjects; hemoglobin denaturation temperature (T_m_^Hb^, (**c**)) and excess heat capacity (c_P_^Hb^, (**d**)); excess heat capacity of Band 3 peak (c_P_^B3^, (**e**)) and the exothermic transition (**f**) as a function of the storage time. Controls have been shown with blue squares, while pathological cells with red dots. The amplitude of the endothermic transition appearing above 75 °C is shown with blue diamond (control) and red triangle (G6PC) in panel (**f**).

The calorimetric profiles from control and G6PD-deficient erythrocytes suffer strong changes during aging, which were followed up to the 33rd day. The calorimetric scans recorded for healthy and G6PD-deficient cells are presented in Figure 7a,b.

In the same way as in our previous study [22], the transition temperature, T_m_^Hb^, and the excess heat capacity, c_P_^Hb^, of Hb gradually decreased during the aging of healthy cells.

For the entire monitoring period of 33 days, T_m_^Hb^ was lower for the pathological cells (Figure 7c), while c_P_^Hb^ values differed significantly from the control values only after 20 days of aging (Figure 7d). Concerning the peak associated with band 3 (B3) proteins, T_m_^B3^ did not change for both samples; however, c_P_^B3^ decreased dramatically after 5 days of aging (Figure 7e). Yet, it should be noted that the shift of the Hb transition toward lower temperatures after 15 days of storage obstructs the B3 transition and thus its reliable analysis. A notable difference between the healthy and G6PD-deficient cells along the aging process was found for the behavior of the exothermal transition, attributed to post-denaturation Hb aggregation. In fresh cells, both control and favic, it occurred at 76 °C. In the course of aging, its amplitude, c_P_^exo^, was stable up to the 12th day for healthy erythrocytes, followed by a decrease accompanied by a shift in T_m_^exo^ towards 80 °C on the 19th day (Figure 7f). Finally, this transition disappeared upon further storage, and this is indicative of a lack of the aggregation process following the Hb denaturation. For G6PD-deficient cells, the amplitude of the exothermic transition sharply decreased and disappeared much faster, i.e., after 10 days of storage, than for the healthy cells. For both cell types, an endothermic transition located at 79 °C appeared after 26 and 15 days of aging for healthy and pathological cells, respectively, indicating the presence of the Hb fraction with more stable conformation than the one denaturing at ca. 70 °C. Prolonged storage of 33 days for healthy and 19 days for favism cells resulted in a single calorimetric transition (Figure 7a,b), suggesting that either only one Hb conformational state is denaturing in a single transition, or the thermal transitions of the different Hb conformations overlap.

**Figure 8:**
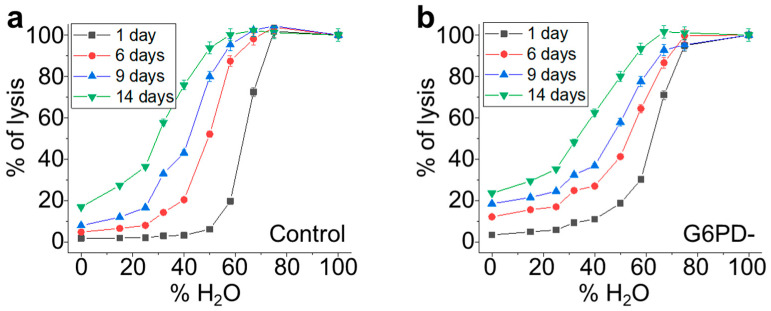
Fragility curves in conditions of osmotic stress measured for control (panel (**a**)) and G6PD-defective (panel (**b**)) erythrocytes. For each sample, the curves have been measured at different aging times from 1 day up to 14 days of aging. All data points are reported with their SD error bars.

In order to directly investigate the mechanical resistance of the cells, we performed osmotic stress measurements by exposing the erythrocytes to solutions at different percentages of H_2_O (i.e., lowered values of osmolarity). The resulting osmolarity curves (the cell’s fragility curves), are depicted in Figure 8a for the control samples and 9b for the G6PD-deficient sample and show the distribution of the RBCs’ mechanical resistance at different aging times. The same osmolarity curves have also been collected for rejuvenated samples in order to quantitatively evaluate the capacity of the cells to recover their mechanical performance following the rejuvenation process.

The two trends have similarities: With increasing aging, all the RBCs become less resistant to osmotic pressure but, in the favism case, the initial sigmoidal shape is maintained, while it progressively changes in the control sample.

To obtain quantitative information on the RBC populations beneath these fragility curves, we performed a fit of the data using a sigmoidal curve according to Equation (2) (see Materials and Methods section).

**Figure 9:**
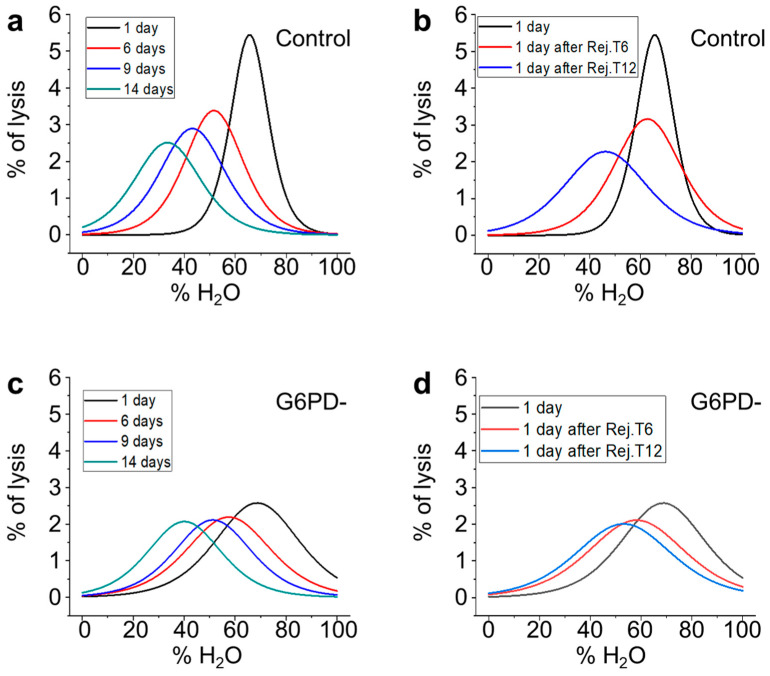
Osmotic resistance curves measured along the erythrocytes aging. Panel (**a**,**c**): time evolution of the RBCs from the control and G6PD defective samples, respectively. Panel (**b**,**d**): effect of the rejuvenation procedures on the control and G6PD defective RBCs, respectively.

In Figure 9, we report the first derivative of these sigmoidal curves, which result in pseudo-Gaussian distributions, which represent the population of RBCs that is beneath the fragility curves. According to Formula (2), in these curves, the x value of the peak is x_0_, its y value is equal to the slope, and the width of the Gaussian is directly related to dx.

With these premises, the graphs shown in Figure 9 describe certain characteristics of the RBCs populations and their evolution over time for the control, G6PD-deficient, and rejuvenated samples. For instance, Figure 9a shows the evolution of the RBCs’ population during aging for the control sample and evidences that, with increasing aging, the population becomes less homogeneous (the max value of the peak decreases), more fragile (the peak moves towards left), and more disperse (the width of the distribution increases).

This process is progressive and continues throughout the aging process. Figure 9b reports the RBC population of the control samples one day after the rejuvenation performed either on day 6 or day 12. It shows that the rejuvenation performed after 6 days reverts x_0_ to a value compatible with the native sample, but the rejuvenated cells result in being less homogeneous than the control after 1 day of aging (black line) and also less homogeneous than the very same population before the rejuvenation procedure (red line of panel a). Similar considerations can also be made for the control sample rejuvenated after 12 days (blue line). Indeed, in this case, the x_0_ value after rejuvenation is lower than the value measured on the initial population after 1 day, while the spread of the population increases, as the width of the curve after rejuvenation is even larger than that of the control samples after 14 days (green curve of panel a).

The data obtained on the G6PD-deficient samples are shown in panels 9c,d. In particular, the time evolution of the sub-populations (see Figure 9c) determines a constant drift towards lower x_0_ values that, differently from the control, is coupled with a very low variation in slope and dx. In Figure 9d, the effect of the rejuvenation in G6PD-defective samples is presented. The data show negligible effects in terms of x_0_ and width associated with rejuvenation after 6 days, while the same procedure performed after 12 days results in a significant recovery of the mechanical resistance (x_0_), although a broadening of the curve (even when compared to the 14-day-aged sample) takes place. Interestingly, in the G6PD samples, no variation in the curves broadening occurs when comparing the samples rejuvenated after 6 or 12 days.

## 3. Discussion

The present study deals with the evaluation of the characteristics and behavior of G6PD-deficient cells as compared to normal erythrocytes.

In the absence of a specific stressor (e.g., fava beans, aspirin, or some antimalaria drugs), the favism, which strictly depends on the presence of defective G6PD, does not show symptoms, and the blood of favism patients is generally assumed to be normal [2,23]. Yet, under exposure to specific stressors, the scenario changes abruptly and severe hemolysis occurs. Typically, the first strong hemolytic crisis removes the weakest and oldest cells from the blood, and subsequent exposure to the same stressor can result in, somehow, less severe symptoms. This seems to suggest that after the removal of a more susceptible fraction of cells, the behavior of the residual population could be different.

Our data provide several insights into this hypothesis. For instance, the observed lysis rate showed marked differences over time between controls and G6PD-deficient cells and such differences became even more evident after a rejuvenation procedure. These differences in the lysis rate are, in fact, explained by the existence, in the G6PD sample, of a significant fraction of cells particularly fragile or otherwise susceptible to lysis. As time increases, and after the extinction of this fraction, different behavior emerges, driven by the residual cells, which appear to be considerably more resistant than the control erythrocytes. This effect is particularly well highlighted by the behavior of rejuvenated cells. Indeed, the rejuvenation process, by definition, consists of an energy reload, which, in all the samples, intrinsically selects only the fraction of RBCs with an active metabolism while the others are lost. In principle, the later the rejuvenation is performed, the larger the fraction of cells lost.

In this framework, our data showed that after rejuvenation performed at 12 days of aging, but not after 6 days, the lysis behavior of G6PD-defective cells evidenced a residual fraction of G6PD-deficient cells, which was much more resistant to lysis than control samples. This surprising behavior demonstrates the existence of a significant fraction of pathological cells that keep their metabolism active whatever the aging duration is. In this sense, it is worth noting that rejuvenation in our experimental conditions was performed at rather long aging times and, consequently, the result is particularly remarkable.

From a morphological point of view, the cells of the G6PD sample evidence a larger number of morphological defects already at shorter aging durations, a circumstance, roughly speaking, comparable to healthy control samples investigated at a more advanced stage of aging. For example, G6PD-defective cells show a significant level of vesiculation already after 1 day of incubation. Vesiculation is known to occur along the lifespan of RBCs, and its biological role is still debated. In particular, it seems that it can play a protective role in vivo [24], while, according to suitable models, it can be related to increased rigidity of the cytoskeleton under an echinocytosis evolution [25]. The AFM data confirm that this latter condition occurs in G6PD-deficient cells, as the percentage of echinocytes is non-negligible already at very short aging times.

Coupling the analysis of the morphological and roughness data allows us to analyze the membrane structure simultaneously. The acquired data evidenced different stages in the maturation of the vesicles and highlighted that the residual membrane (i.e., outside the vesiculating areas) is much smoother and more featureless than in the control cells. This effect can be explained by the occurrence of an extensive structural rearrangement that takes place at the membrane level due to the vesiculation, but also the existence of a re-organization of the skeletal component during this process. This latter phenomenon is detected through the behavior of the membrane roughness whose quantitative value is known to be sensitive to the structural integrity of the cell skeleton [14,17].

In this regard, a previous work [26], using t-BHP as an oxidizing agent, highlighted the occurrence of band 3 proteins’ aggregation and effects on membrane and roughness possibly mediated by Ca^+2^ ions. In this sense, our data, perfectly compatible with a contribution to the skeletal rearrangement arising from anomalies in the distribution of band 3, show that effects on the roughness can be observed even in the absence of extracellular Ca^+2^, suggesting that alternative pathways (or alternative messengers) may play a role in the regulation of the aging dynamics of the membrane-skeleton.

Certainly, the behavior of the membrane roughness measured in the two samples is quite interesting, as G6PD-deficient cells have a peculiar trend during aging, and it is interesting to note the relationship between roughness and intracellular ATP. Indeed, by comparing the trends over time of these parameters (see Figure 5 and Figure 6), a clear analogy can be seen: At the beginning, in the samples with favism, the cells start with a lower supply of ATP and an already altered structural architecture as testified by the low roughness value measured after 1 day. Yet, the subsequent rate of roughness decreasing is approximately 4 times smaller than in normal cells, and this time evolution is also associated with very low consumption of ATP (it is worth reminding that, in our experimental conditions, de novo synthesis of ATP cannot occur).

The relationship between these two cellular parameters is not surprising, based on previous studies [21], including some focused on the correlation between morphological and biochemical characteristics [14,15]. These studies have shown that, among the cellular resources, ATP is the one that can be directly associated with the best anchorage of the skeleton to the overlying plasma membrane. The membrane roughness is, indeed, sensitive to the morphological consequences of these structural links. The biochemical explanation can be provided by the phosphorylation of the cytoskeleton, a PKC-controlled phenomenon that can be activated by ATP (or glucose) shortage [27] and can result in transient or permanent (via caspase activation) dissociation of the cell skeleton from the membrane [28,29].

In this sense, the strong similarity between the membrane roughness and the intracellular ATP trends is very interesting. Indeed, comparing the data in Figure 5 and Figure 6, we note the absolute analogy of the two trends, which confirms the great importance of ATP in controlling and regulating the interactions between the skeleton and the plasma membrane. This clearly underlines the role of ATP in maintaining the structural integrity of the membrane skeleton and, consequently, in preserving the characteristics of elastic deformability in RBCs.

Furthermore, the comparison between the consumption rates of ATP in healthy and pathological RBCs reveals the existence of significant metabolic differences in G6PD-defective cells, which results in a remarkable resistance to cell aging. Indeed, despite an evident difficulty of the pathological cells in ensuring an adequate supply/production of ATP (see data on day 1), these cells are evidently adapted to carry out their essential activities with a lower basal level of ATP and exert a much lower energy consumption than that of the normal cells. As a consequence, while at short aging times the favism cells seem more fragile, morphologically unstable, and vulnerable to environmental stresses (especially to the oxidative ones), at longer aging times, these cells only experience a partial lack of resources and they can much more effectively fight the development of late-aging characteristics.

It is worth stressing that the measured ATP trend is well suited to explaining the super-resilient lysis behavior of G6PD-defective cells observed at long aging times or after the rejuvenation process.

Furthermore, the existence of different metabolic regulations in normal and favism erythrocytes is also suggested by the significant differences observed in the rate of Hb reoxidation following the rejuvenation procedure. It is well known that the balance between the production of ATP and reducing power is metabolically regulated through the glycolysis or pentose phosphate pathways (PPP) in order to privilege the first or second according to the cell needs. Yet, in G6PD-deficient cells, this balance is intrinsically dysregulated [30] and depletion of reducing power, especially in terms of NADPH, takes place. This, however, mostly affects the defense against H_2_O_2_ rather than the Fe^III^-Hb control, as this latter is prevalently ensured by the NADH system, which, on the other hand, is essentially produced simultaneously with the ATP in glycolysis [31]. In our case, however, according to the measurement of the intracellular ATP, we do not expect a high production of NADH, a circumstance confirmed by the faster oxidation kinetics of native Hb. Yet, the Hb reoxidation kinetics after rejuvenation showed the existence of sharp qualitative differences between control and pathological cells: A quasi-linear behavior has been observed in pathological senescent cells instead of kinetics, which accelerates with time, as observed in the senescent controls. The most straightforward explanation for this phenomenon is that the two biosystems have a different use of the cell resources, with the pathological cells that have a higher amount of reducing power available, at least in those extreme conditions. Thus, a deeper metabolic regulation, regarding not only the consumption of ATP but also the use of reducing power, can be hypothesized to take place in G6PD-deficient cells.

To better understand the behavior of pathological RBCs, it is certainly very important to consider what the structural and functional characteristics of these cells are compared to normal erythrocytes and, possibly, how they can be associated with different metabolic behaviors. In this context, the results of DCS and the resistance to osmotic stresses can provide useful information on cell characteristics and behavior.

All the DSC features indicate strong changes in the conformation of erythrocyte proteins along the aging path. Overall, indeed, Hb and likely the Band 3 protein are more stable against a thermal challenge along the aging of erythrocytes in the control samples compared to G6PD-defective cells.

The dynamics of the main Hb transition during aging are clearly different in the two samples, and it is evident that the aging-related Hb transformation into its thermally unstable conformation occurs faster in G6PD-defective than in control RBCs. Interestingly, it is known that defective denatured Hb binds to the band 3 protein, providing a signal that can promote cell removal from circulation in vivo.

Thus, the DSC data clearly indicate greater protein and structural instability in the pathological cells. This information agrees with the faster rate of Hb oxidation, as well as with the AFM and roughness data, which already evidenced the existence of non-negligible morphological alteration in the erythrocytes after 1 day of aging. Therefore, in agreement with the DSC data, the fact that the first marker of aging occurs sooner in G6PD-defective erythrocytes could depend on the lower intrinsic stability of some of their cellular components. The DCS data indicate that the aging of RBCs accelerated the thermal destabilization of Hb, and the disappearance of the transition related to post-denaturation Hb aggregation for subjects with the G6PC mutation compared to healthy controls. Such a significant effect on Hb results in a different denaturation dynamic and the distinct evolution of the DSC scan for controls and G6PD-defective cells along the aging path.

In this regard, since no Hb structural defects are usually associated with G6PD deficiency (neither in the literature nor in the specific case study), its lower structural stability should not depend on intrinsic protein differences but rather on defects associated with the cellular micro-environment, which, in the G6PD-defective case, is known to be more susceptible to oxidative stresses. In this sense, the faster and more severe oxidation rates of hemoglobin measured in our study (Figure 1) could actually play a role. In a larger view, Hb can act as a trigger for the later stage of the aging pathway [32] by connecting its denaturation state with effects at the membrane level, such as the detachment of the skeleton from the membrane and the production of Heinz bodies (the latter is known to occur in vivo following a hemolytic crisis). In this pattern, however, a very important mitigating (or even protective) role can be played by the higher ATP concentration that we measured (Figure 6) at long aging times in the G6PD-defective RBCs, which could counteract the disengagement of the membrane from the skeleton.

Thus, a dual pathway can be speculated to occur in favism cells, with one path that, at medium aging times, increases the cell alteration because of the Hb instability and the other path that counteracts the development of skeletal defects thanks to the larger availability of ATP at longer aging times. The morphological consequences of this biochemical signaling are well underlined by the AFM data. In particular, the initial worse morphological condition of G6PD-deficient cells dominated by spiculated morphologies and their very slow degradative evolution along the aging path are results that perfectly fit this picture.

Osmotic stress is a direct probe of the mechanical resistance of cells. Our approach focused on the investigation of the samples’ behavior based on the conversion of the osmosis-induced lysis curves (Figure 8) into a pseudo-Gaussian distribution (Figure 9), whose parameters indicate the overall cells’ mechanical properties and the presence of a different subpopulation. An advantage of this approach is that it provides the opportunity to analyze how a given distribution of mechanical resistance evolves over time as a function of aging.

The data measured in the controls and G6PD-deficient samples analyzed with native aging and on samples submitted to rejuvenation after 6 or 12 days showed several important differences. The first observation is that, compared to the controls, the pathological RBCs have, in the majority of the experimental conditions, a higher osmotic resistance as measured by x_0_ (see Figure 9). Furthermore, other intriguing differences involve the homogeneity of the native samples and their time evolution.

Indeed, at the beginning of aging (day 1), the controls are very homogeneous (narrow and sharp curve) and the effect of aging, in addition to the reduction in osmotic resistance, determines a progressive broadening of the curve, thus revealing the existence of subpopulations, which lose mechanical resistance faster than average while others lose it less quickly than average. In contrast, in the G6PD-deficient sample, aging only causes a rigid shift of the curves toward lower mean resistance values. This trend indicates that all the sub-populations aged over the same time scale, resulting in a constant spread of the overall population over time (dx and slope are essentially constant). Furthermore, in G6PD-defective cells, the curve after 1 day of aging is already very wide, highlighting the existence of a variety of subpopulations already in the native G6PD sample.

The fact that, in the controls, there are subpopulations that age with different speeds indicates that, as the environmental and metabolic cell status become harsher, the RBCs might face a critical condition (very likely the lack of resources) resulting in dramatic changes in the cellular properties and metabolism. Very remarkably, and in agreement with what we observed for the ATP consumption and the Hb oxidation rate, such critical conditions are never really experienced by the G6PD-deficient cells. As a consequence, the aging of these cells appears to be a purely chronological phenomenon with limited consequences on the cell properties.

This is a significant change in paradigm for the entire picture we have of the aging phenomenon.

Other interesting considerations arise from the comparison of the rejuvenation pathways. Indeed, in the controls, the rejuvenation is effective in improving the mechanical resistance of RBCs (see the red curves in Figure 9a,b, and blue vs. blue and green in the same panels), but it also determines a broadening of the curves that testifies a certain, expected stress, which is induced in the metabolic cell machinery by the rejuvenation procedure. The behavior of G6PD-deficient cells in the same conditions is sharply different: The rejuvenation after 6 days is nearly ineffective on the osmotic resistance and the curve broadening, while the rejuvenation after 12 days results in a significant recovery of the osmotic resistance.

This surprising result has a clear interpretation that confirms what is observed in the cell lysis curve under rejuvenation. Indeed, as already mentioned, each rejuvenation restores the resource only in the RBCs that are still metabolically active while the other cells are lost. In this view, the behavior of the osmotic resistance following rejuvenation suggests that in the G6PD-deficient sample, with long aging times, the residual population of cells is constituted by the majority of cells that have not really experienced a critical lack of resources. As a consequence, these super-resilient cells in the pathological sample can actively counteract the environmental stimuli and, in any case, show superior functional performances compared to the controls.

Finally, the analysis of the broadening of the curves after revitalization (Figure 9b,d) has evidenced that, differently from the controls, the pathological curves have a nearly constant width even at very high aging times. This observation has interesting consequences as it means that under the pathological curves, there are fewer available configurations for the cells. Obviously, as the present point of view concerns mechanical resistance, the sense of available configuration refers to structural variabilities that have consequences in terms of mechanical properties. In speculative terms, this could mean that there are alterations of the membrane skeleton that do not occur in pathological cells, or that the ion imbalance (ATP-dependent) in the long run is less severe in these cells compared to controls.

A concluding remark on the significance of the present findings in the general context of favism pathology is in order.

Overall, our data highlighted several peculiarities of G6PD-deficient RBCs, which would not be detectable if they were not studied during aging. In fact, an analysis performed at day 0 (i.e., on native cells) would essentially reveal morphologic-structural defects associated with higher cellular fragility and vulnerability to oxidative stress. Yet, thanks to the analysis of the time evolution of the cells’ parameters during aging, it has been possible to highlight a more complex pattern in which the evolution of the biochemical, morphological, and biophysical characteristics is interconnected and coherent. In our interpretation, the entire pattern arises from the existence of a peculiar cellular metabolic regulation (or metabolic adaptation), which determines a strong reduction in ATP consumption under stress conditions (i.e., starvation and associated disturbances).

In this framework, it would be interesting if this effect is not limited to the special case investigated but rather is associated with a general phenomenon of metabolic adaptation taking place in the erythrocytes from favism patients as a group (at least in the presence of certain types of G6PD mutations). A formal answer to this question, however, is not the aim of this work. Indeed, drawing more general conclusions on favism would certainly require the analysis of a larger number of patients, the evaluation of parameters such as sex, age, and clinical symptoms, and the performance of dedicated clinical investigations. However, it is interesting to underline that the present hypothesis of a peculiar metabolic regulation occurring in favism patients is supported by independent evidence. For instance, some papers reported different metabolic regulation after mild oxidative stress [33] and surprisingly good storage performances of G6PD-deficient cells in blood bank conditions [34], a circumstance that, notwithstanding a different buffer employed, is in full agreement with our findings.

## 4. Materials and Methods

### 4.1. Sample Preparation

Blood samples were obtained from one healthy subject and one favism patient, with mild symptoms, suffering from the Mediterranean variant of the G6PD. Both the donors were of the same sex (male), race (Caucasian), and age. The blood samples were collected through venipuncture into Vacutainers (Becton-Dickinson, Franklin Lakes, NJ, USA) and were immediately diluted 2-fold in a calcium- and glucose-free buffer solution (10 mM sodium phosphate, 140 mM NaCl, EDTA 1 mM as the anticoagulant, and adjusted to pH 7,4 with NaOH), and then centrifuged for 10 min at 3000 rpm at 4 °C. The platelet-rich supernatant and the white layer on top of the pellet (i.e., leukocytes) were discarded, while an aliquot of the supernatant plasma was stored at 4 °C for the AFM smears. After 4 cycles of re-suspension and washing in the same phosphate buffer, the isolated RBCs were stored under sterile conditions at room temperature (20 ± 1 °C) at a 20% volume fraction in the buffer solution. A protease inhibitor (phenyl-methyl-sulfonyl-fluoride, 1 mM) was added to the RBC solutions in order to avoid proteolytic degradation during aging. In order to accelerate the time-scale of 1in vitro aging, the entire incubation was carried out in the absence of glucose and extracellular calcium.

All reagents were of analytical grade and were purchased from Sigma-Aldrich (St Louis, MO, USA).

### 4.2. Spectrometer Measurements (Percentage of Lysis and Hb Oxidation)

To evaluate the progression of the aging phenomena, we monitored the percentage of cells that lyse and the oxidation state of the intracellular hemoglobin using a spectrophotometric method. The spectra of each sample were acquired through a double-beam spectrophotometer Jasco V-630 (from Jasco Co., Tokyo, Japan) in the range of 350–700 nm where the three characteristic peaks of Hb appear. The Soret peak at approximately 414 nm has been used for the evaluation, according to the following procedure: (i) For extracellular Hb, 60 μL of the cellular solution was taken and diluted 1:20 in the buffer solution, then centrifuged at 3200 rpm for 12 min in order to remove the intact cells from the solution. An aliquot of 800 μL of the supernatant was taken and diluted 1:1 in buffer and then measured. The wavelength of the Soret Peak was used to evaluate the oxidation state of extracellular Hb. (ii) For intracellular Hb, 6 μL of the cellular solution was diluted 1:200 in bi-distilled water to completely lyse the cells. Then the sample was centrifuged at 3200 rpm for 12 min, and 1 mL of the supernatant was further centrifuged at 11000 rpm for 11 min to remove the cellular debris from the solution. An aliquot of 800 μL of the supernatant was taken and diluted 1:1 in bi-distilled water and then measured. The position (wavelength) of the Soret peak has been used to evaluate the oxidation state of intracellular Hb. (iii) From the above-mentioned spectra, the percentage of lysis was calculated from the ratio between the values of absorbance of the extracellular vs. intracellular (i.e., 100% lysis) spectra.

Moreover, our experimental procedures ensured that the aliquots measured did not contain free cells, bilirubin, and plasma, and for these reasons, we did not need to correct the spectra baselines prior to data extraction.

### 4.3. ATP

The measurement of the intracellular ATP levels was performed every 2–3 days by means of the kit “Cell Titer-Glo Luminescent Call Viability Assay” (Promega, Madison, WI, USA) and a Wallac 1420 Victor3 V plate reader (Perkin-Elmer, Waltham, MA, USA). The procedure to prepare and measure each sample was as follows: (i) A 200 μL aliquot of the sample was diluted 1:1 in buffer solution and then centrifuged at 3200 rpm for 12 min, and then all the supernatant was carefully removed and the remaining pellet was diluted 1:5 (*v*/*v* ratio) in perchloric acid 0,6 M; it was then gently mixed and centrifuged at 8000 rpm for 8 min in order to remove all the denatured proteins from the sample. An 80 μL aliquot of the supernatant was neutralized during 1 h of incubation in an ice bath in the presence of 6:1 potassium carbonate 2,5 M. (ii) A 240 μL aliquot of the supernatant was centrifuged at 11000 rpm for 11 min in order to eliminate any residual pellet and an aliquot of 200 μL of this supernatant was used for the measurement of the intracellular ATP. (iii) For each sample, two aliquots of 65 μL each were measured in two different 96 multiwall plates by adding 65 μL of the reagent and, according to the recommendations of the manufacturer, incubating the solution for 10 min in a dark environment.

All reagents were of analytical grade and were purchased from Sigma-Aldrich (St Louis, MO, USA).

For the intracellular ATP measurements, the pellet was diluted 1:5 (*v*/*v* ratio) in perchloric acid 0.6 M, gently mixed, and then centrifuged at 8000 rpm for 8 min to remove all the denatured proteins from the sample. A fraction of the supernatant (typically 80 μL) was neutralized in a new vial by adding 6:1 (*v*/*v* ratio) potassium carbonate 2,5 M followed by one hour of incubation in an ice bath. Next, 240 μL of the supernatant was centrifuged at 11,000 rpm for 11 min to eliminate any residual pellet. A 200 μL fraction of this supernatant was used for the measurement of the intracellular ATP

The measurements were performed after 20, 30, and 40 min after the addition of the reagent. To correlate the counts with the concentration of ATP, each measured plate contained a standard calibration series of ATP that was freshly prepared before the measurements by the progressive dilution of a batch of the 16.5 M ATP solution (Sigma-Aldrich, St Louis, MO, USA). The obtained results were corrected according to the percentage of cell lysis to associate the values with the effective number of cells in the solution. Moreover, to grant the cross-correlation with different experimental runs, the obtained values were normalized to a standard RBC solution with 45% hematocrit.

### 4.4. Sample’s Smears Preparation

The smears used for the AFM characterizations were performed in duplicate every 2–3 days starting from a plasma-enriched solution (15 μL of sample plus 15 μL of plasma) that was manually smeared onto a commercial poly-L-lysine-coated glass slide (Menzel-Glaser, Braunschweig, Germany) and then air-dried [35]. The addition of plasma ensures a homogeneous dispersion of the cells on the entire glass slide, and the quantity used (5 μL for each smear) grants an optimal density for AFM measurements. The plasma was stored at 4 °C but was allowed to reach room temperature prior to its use in order to avoid thermal stresses to the cells. We verified that such smears, if properly stored, can last un-modified for years.

### 4.5. AFM Characterizations (High-Resolution Imaging and Membrane Roughness)

The AFM images were collected using a home-designed microscope, working at room temperature and constant 30% relative humidity. The measurements were performed in contact mode using Silicon Nitride Veeco MSCT probes (Veeco, Camarillo, CA, USA) with a 0.03 N/m elastic constant, asymmetric pyramidal shape, and nominal tip radius of 10 nm, maintaining the maximum force between the tip and the sample below 1 nN. High-resolution images used to measure the membrane’s roughness, were collected at a scanning speed of 3–4 s/row, and the reproducibility of data was carefully tested.

The surface roughness of erythrocyte’s plasma membrane has been proven to be a sensitive parameter to evaluate the structural integrity of the RBCs and, as a consequence, the mechanical support that the cell skeleton can exert. The methodology we employed here has been slightly modified compared to the previous paper on this subject in order to improve the determination of the surface roughness and reduce the experimental error. A detailed discussion of the methodology employed for the measurement and analysis is reported in references [14,15], while general advantages and peculiarities in the use of membrane roughness are reported in [17]. The present method employs the free software Gwyddion (www.gwyddion.net accessed on 1 February 2020) and consists of selecting several (typically more than 10) sampling areas on the cell membrane, all of a fixed 1 × 1 μm size. After background subtraction, all the residual morphological components were removed by fitting the X and Y axes with a high-grade polynomial. The 7th grade was used, although 8th grades could have been used with similar effectiveness and very limited quantitative differences.

After fitting, the surface roughness was measured using the formula:

(1) $$R_{\mathrm{rms}}=\frac{1}{\left(N-1\right)}\sqrt{\sum_{1}^{N}{\left(X_{i}-X_{m}\right)}^{2}}$$

where N is the total number of data points, Xi is the height of the i-th point, and Xm is the mean height value.

### 4.6. Rejuvenation Procedure

The rejuvenation procedure (also named revitalization) was performed at selected aging times according to a modified De Venuto protocol [36]. The sample, an entire vial, was centrifuged at 3000 rpm for 12 min, the supernatant was discarded, and the remaining cells were diluted 1:5 in the rejuvenation buffer, composed of 10 mM inosine, 10 mM pyruvate, 75 mM sodium phosphate, 23 mM NaCl, 5 mM NaOH, and pH 7.4 (Sigma-Aldrich, St Louis, MO, USA), and incubated for 3 h at 37 °C. After two subsequent washing procedures (in standard buffer, 3000 rpm, 12 min each passage), the cells were resuspended in the standard buffer at a final hematocrit of 20%. A 200 μL aliquot of the sample was taken before and after the rejuvenation procedure to measure the ATP content. The rationale of this procedure consists of providing the cells with the raw materials needed to build up their own ATP levels, which means that only the cells with active metabolic machinery will be able to increase their ATP content; in this way, we were able to obtain insight into the metabolic integrity of the cells.

### 4.7. Osmolar Stress

An aliquot of the sample (30 μL) was taken and immediately diluted 40-fold in buffer solutions at different percentages of H_2_O (namely, 15%, 25%, 33%, 40%, 50%, 58%, 67%, and 75%) in order to expose the cells to different osmolarities. After a few minutes, the cells were centrifuged at 3200 rpm for 12 min and 1 mL of the supernatant was taken, diluted 1:1, and measured with a spectrophotometer. The value of standard lysis measured on the very same day was taken as the value for the 100% buffer (i.e., 0% of H_2_O) while the sample at 100% H_2_O was prepared by diluting 6 μL of the RBC solution in 2 mL of ultrapure water, centrifuging it at 3200 rpm for 12 min, and then diluting it 1:1 prior to measurement with the spectrophotometer.

**Figure 10:**
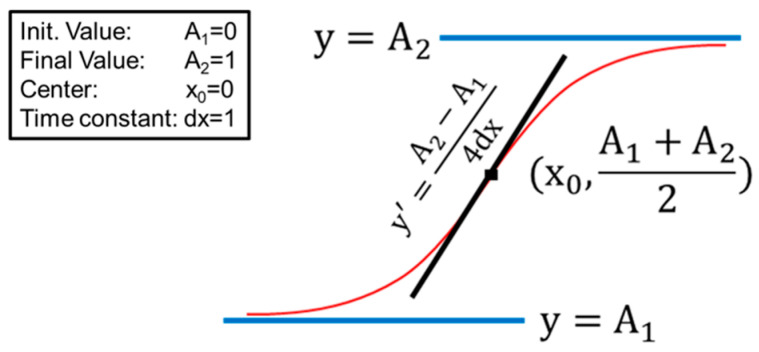
Sketch of the sigmoidal curve used for the analysis of the fragility curves of RBCs under osmotic stress, as derived from Equation (2).

The obtained fragility curves were subsequently fitted using a sigmoidal curve following Equation (2) and Figure 10. In Equation (2), the curve is described by the following parameters: x_0_ is the amount of H_2_O required for 50% lysis; the slope of the sigmoidal curve in x = x_0_ measures the steepness of the curve; (iii) dx is directly related to the spread of the sigmoidal curve. 

To ensure good agreement between the fitting and the experimental data, we carefully checked the compatibility of the fitting parameters A1 and A2 with the initial lysis value (i.e., the standard lysis measured with 100% buffer) and the 100% lysis, respectively. In all the performed fits, we could confirm this compatibility. Moreover, to visually inspect the population that stands beneath these fragility curves, we performed the first derivative of the fitting curves and plotted them against the percentage of H_2_O. The resulting pseudo-Gaussian single-peak curves immediately showed the values of the fitting procedure, where the x coordinate of the peak was equal to x_0_ and the y value of the peak was equal to the slope, while the width at mean height was approximately 3.5 times the dx.

(2) $$y=\frac{A_{1}-A_{2}}{1+e^{(x-x_{0)/\mathrm{dx}}}}+A_{2}$$

### 4.8. Differential Scanning Calorimetry (DSC)

The calorimetric scans of RBCs from the healthy control and the subject with the G6PC mutation were recorded in the range of 30–90 °C with a scanning rate of 1 °C/min using the DASM 4 microcalorimeter (from Privalov, Puschino, Russia). Prior to DSC measurements, RBCs were washed twice in 10 mM PBS buffer containing 1 mM EDTA and resuspended to a final concentration of 8 mg Hb/mL in the same buffer.

Data were analyzed by the Origin software routine and the thermodynamic parameters: Temperature, T_m_, and excess heat capacity, c_P_, of the thermal transitions corresponding to the Band 3 protein (T_m_^B3^ and c_P_^B3^, respectively), Hb (T_m_^Hb^ and c_P_^Hb^, respectively), the exothermic transition (T_m_^exo^ and c_P_^exo^, respectively), and the peak at 80 °C in aged cells were determined.

### 4.9. Data Analysis

All the data analyses were performed using the software package “Origin 8”, while the AFM images were analyzed through the freely available software Gwyddion (www.gwyddion.net accessed on 1 February 2020) [37].

All the experiments reported in the paper were repeated at least three times.

The statistical analysis has been performed using a one-way ANOVA test.

## 5. Conclusions and Future Perspectives

The comparison between the aging pattern of normal erythrocytes with that of cells deficient in G6PD, a defect pathologically associated with favism, highlighted significant qualitative and quantitative differences. In particular, favism erythrocytes, compared to normal cells, are initially more fragile, more exposed to oxidative stress, and, already in pseudo-native conditions (e.g., 1 day of aging), morphologically associated with anomalies such as shape alterations, the presence of micro-vesicles, and defects of the membrane–cytoskeleton interaction. However, as the aging time increases, the progression of aging phenomena in G6PD-cells is much slower, and at long aging times (>10–12 days), these same cells show properties of greater resilience than the normal cells. This is evidenced, for example, by the cell lysis and the osmotic resistance at long aging times (especially in revitalized samples), and by the oxidation kinetics of Hb and by the AFM data, both in terms of morphology and membrane roughness.

The entire pattern of the observed biochemical and biophysical data is explained, in the case of favism, by the existence of mechanisms of metabolic regulation that cause an extremely reduced consumption of ATP, compared to normal cells, during aging in stress conditions (starvation). In this sense, the cells of the G6PD-deficient sample were found to be metabolically more ready to endure extreme environmental stress than normal erythrocytes.

Understanding whether or not this peculiar metabolic regulation is a general mechanism occurring in favism patients and, eventually, what the molecular mechanisms involved in the regulation are, is not the aim of the present paper. However, the answer to this question provides an extremely interesting future perspective for the present study. In particular, understanding the importance of different proteomics in the structural and functional differences observed could be highly desirable, as well as investigating the role played by the older and younger RBCs present in the blood at a given time [38,39]. In these contexts, in particular, the capabilities of molecular recognition imaging of AFM to identify selected biomolecules using a specific tip sensor [40] could potentially play a role.

## Data Availability

All the data presented in this study are freely available from the corresponding author upon reasonable request.
